# Shrimp Allergy—Distinct Allergen Sensitization Profiles Between Intercontinental Cohorts

**DOI:** 10.1111/all.16662

**Published:** 2025-07-18

**Authors:** Shaymaviswanathan Karnaneedi, Sara Anvari, Shea Brunner, Karen S. Tuano, Brenda Bin Su, Saachi Hira, Sahel Heidari, Diamond Hira, Carla M. Davis, Andreas L. Lopata

**Affiliations:** ^1^ Molecular Allergy Research Laboratory College of Science and Engineering, James Cook University Townsville QLD Australia; ^2^ Australian Institute of Tropical Health and Medicine James Cook University Townsville QLD Australia; ^3^ Centre for Food and Allergy Research Murdoch Children's Research Institute Melbourne Victoria Australia; ^4^ Baylor College of Medicine, Texas Children's Hospital Department of Pediatrics, Division of Immunology, Allergy and Retrovirology Houston Texas USA; ^5^ Texas Children's Hospital, William T. Shearer Center for Human Immunobiology Houston Texas USA; ^6^ Allergy Medical Centre Townsville QLD Australia; ^7^ Tropical Futures Institute, James Cook University Singapore Singapore

**Keywords:** hemocyanin, igE antibody, immunotherapy, shrimp allergy, tropomyosin

**Abbreviations**
AU: Australia
HC: Hemocyanin
MLC: myosin light chain‐2
PA: *Penaeus aztecus*/Brown shrimp
PM: *Penaeus monodon*/Black tiger shrimp
PV: *Penaeus vannamei*/Vannamei shrimp
SCP: sarcoplasmic calcium‐binding protein
TM: Tropomyosin
US: United States of America

To the Editor,

Shellfish allergy, particularly to shrimp, is a significant cause of food‐induced anaphylaxis, affecting about 3% of adults and 1.3% of children worldwide; and up to 7.7% in the Asia Pacific region [1]. This allergy is typically lifelong, with up to 90% of affected individuals retaining their sensitivity into adulthood [1]. The allergens responsible for shellfish allergies are proteins such as tropomyosin, arginine kinase, myosin light chain, sarcoplasmic calcium‐binding protein, hemocyanin, and others [2]. Shrimps, particularly the Black Tiger ( *Penaeus monodon* ; PM), Brown ( *Penaeus aztecus* ; PA), and Vannamei (*Litopenaeus/Penaeus vannamei*; PV), are common sources of crustacean allergens in globally consumed species. Despite diagnostic and therapeutic advancements, many studies focus on the general cross‐reactivity of shellfish allergens, with fewer investigating species‐specific IgE‐binding patterns [3, 4]. This study explores these patterns in subjects from Australia (AU) and the United States (US) to improve diagnostics and treatments for shrimp allergies.

Three shrimp species, PM (frozen), PA (powder), and PV (frozen), were processed to extract proteins, either raw or heated (boiling for 10 min) to simulate cooking. The proteins were analyzed for their IgE‐binding capacity using immunoblotting (Figures S1, S2) and mass spectrometry (Table S1) to identify which specific allergens were recognized in shrimp‐allergic individuals, providing insights into how different shrimp species trigger allergic reactions in diverse populations.

This study included 30 shellfish‐allergic subjects from both Australia and the United States, who gave written informed consent, with ethics approval (H4313/H6829). (Table S2) Both groups exhibited typical allergic symptoms after shrimp consumption, with the AU cohort having a higher prevalence of asthma, allergic rhinitis, and atopic dermatitis. PM and PV are highly consumed in Australia, while PA is in the United States but not available in Australia. The study found significant differences in the IgE‐binding profiles of the three shrimp species. For raw PM shrimp, all AU subjects showed IgE binding to the 35‐38 kDa protein, identified as tropomyosin, in contrast to US subjects. PA produced a similar binding profile to PM in the AU cohort, but more frequent binding to the 75 kDa protein, identified as hemocyanin. The US subjects exhibited the most consistent binding to PV allergens, with binding to tropomyosin and hemocyanin prevalent in many subjects. (Figure 1).

**FIGURE 1:**
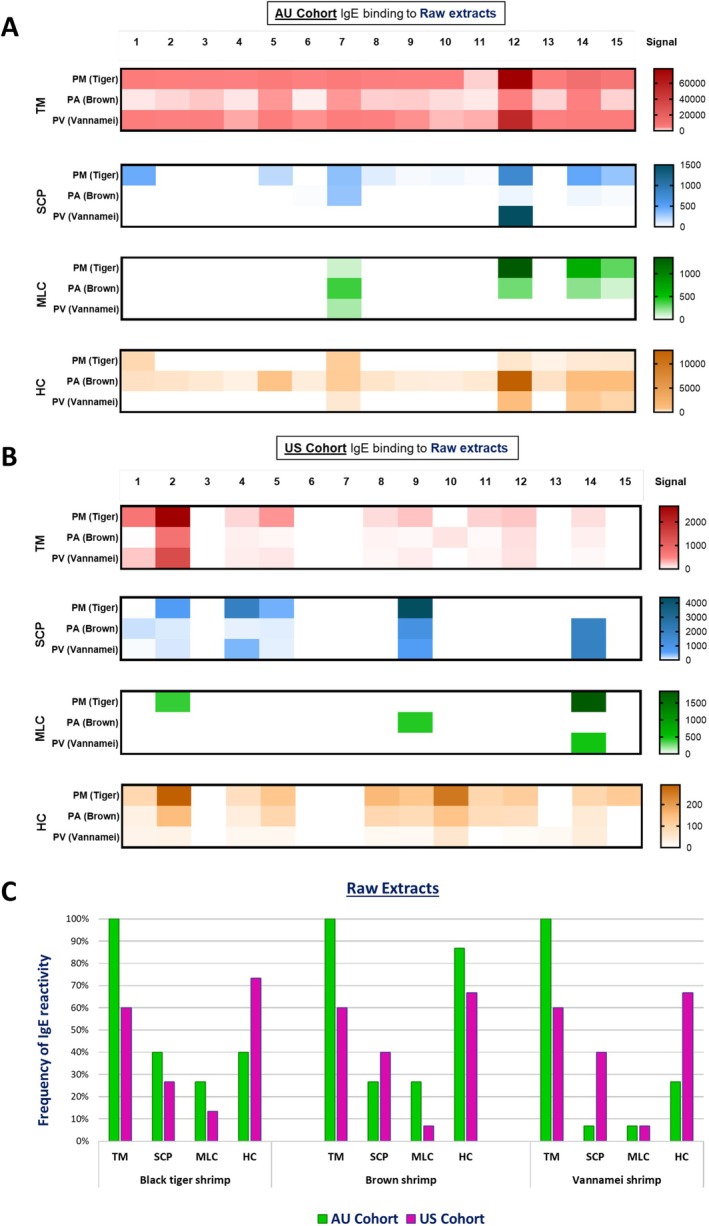
Heatmap using densitometric analysis (A, B) and calculated frequency (C) of in vitro sIgE binding to tropomyosin (TM), sarcoplasmic calcium‐binding protein (SCP), myosin light chain 2 (MLC), and hemocyanin (HC) from raw extracts of Black Tiger shrimp, Brown shrimp, and Vannamei shrimp, using sera from Australian (AU, green) and American (US, pink) shellfish‐allergic cohort. Numbers 1–15 correspond to patients in each cohort.

Heating shrimp proteins reduced, as expected, the overall number of IgE‐binding proteins, particularly in the AU cohort, which showed increased binding to sarcoplasmic calcium‐binding protein (SCP) and myosin light chain (MLC) after heating, compared to raw extracts. For the US cohort, there was less variation in binding between raw and heated extracts. (Figure 2).

**FIGURE 2:**
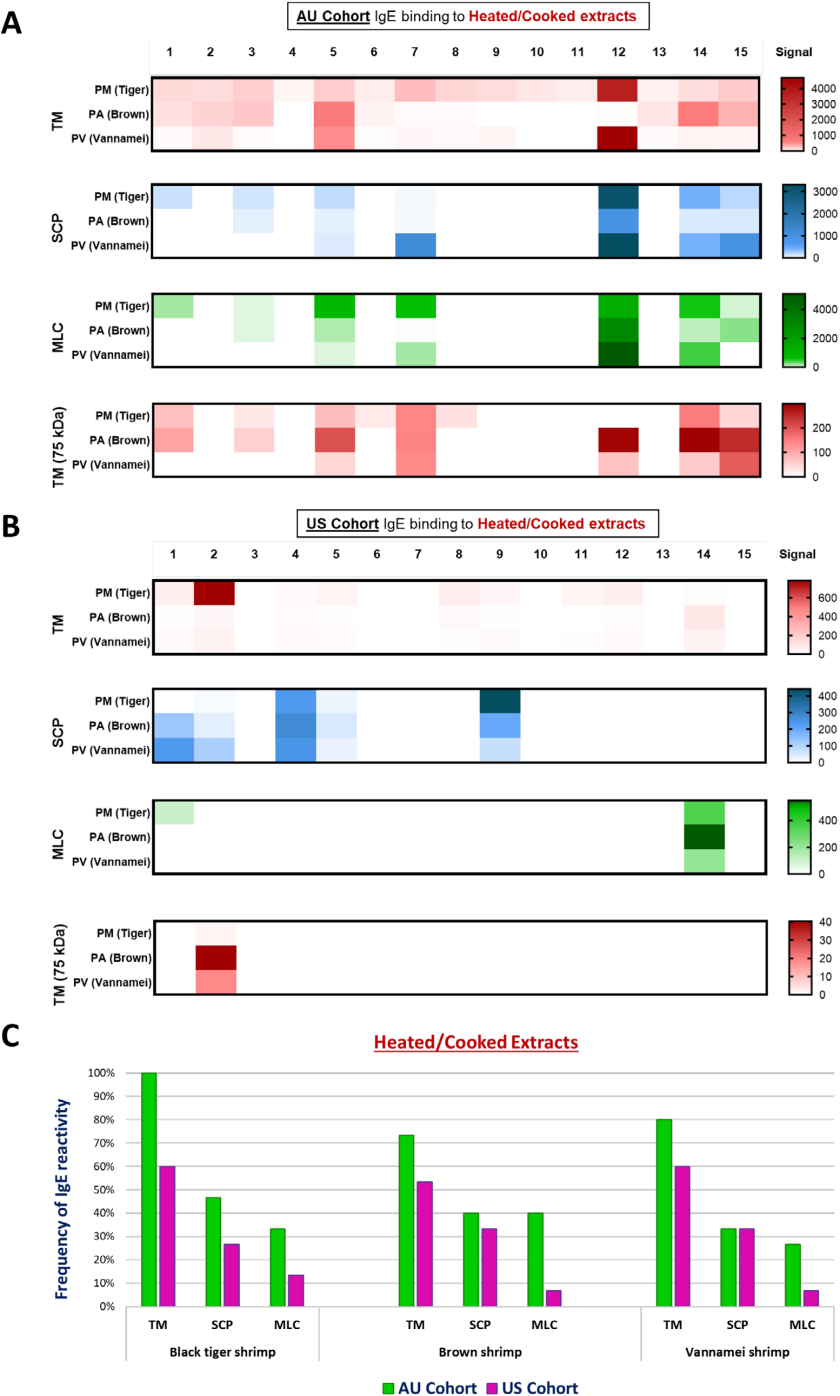
Heatmap using densitometric analysis (A, B) and calculated frequency (C) of in vitro sIgE binding to tropomyosin (TM), sarcoplasmic calcium‐binding protein (SCP), and myosin light chain 2 (MLC) from heated/cooked extracts of Black Tiger shrimp, Brown shrimp and Vannamei shrimp, using sera from Australian (AU, green) and American (US, pink) shellfish‐allergic cohort. Numbers 1–15 correspond to patients in each cohort.

Inhibition assays in a subset of patients using purified recombinant allergens from PV (tropomyosin, SCP, and MLC) revealed that tropomyosin could block IgE binding to the major 35–38 kDa band in PM shrimp, but SCP and MLC only partially inhibited binding to their respective bands and species‐specific reactivity (Figure S3).

This study reveals variability in IgE‐binding profiles to different shrimp species among shellfish‐allergic subjects from Australia and the United States. Tropomyosin, being the major allergen, was consistently recognized by all subjects, though its IgE‐binding pattern varied slightly between species. This variability is possibly attributed to different isoforms or epitopes of tropomyosin, particularly in PA and PV [5]. Hemocyanin, SCP, and MLC showed significant cross‐species variations in their IgE‐binding profiles, which different isoforms or molecular structures could explain [6, 7]. Interestingly, we observed IgE sensitization to shrimp allergens that the cohort had likely never encountered. Specifically, AU subjects were sensitized to hemocyanin allergen from PA but not from shrimps they usually consume. This evidence confirms the importance of tropomyosin, while also highlighting the necessity of considering additional allergens critical for the development of more precise diagnostic tools and targeted immunotherapy for shrimp allergies.

These results suggest that personalized shrimp allergy diagnostics, with focus on key allergens (tropomyosin, hemocyanin, SCP, and MLC), while considering their heat stability in both raw and cooked shrimp, could enhance diagnosis and treatment. This study highlights species‐specific IgE‐binding patterns and individual variability, underscoring the need for further research on allergen isoforms to develop globally relevant crustacean immunotherapy and ultimately optimize patient outcomes.

## Author Contributions

S.K. collected the data, performed analysis, and wrote the manuscript. S.A. contributed analysis tools, performed the analysis, and wrote the manuscript. S.B. contributed data and analysis tools. K.S.T. collected the data. B.B.S. contributed analysis tools. S.H. collected the data. S.H. performed the analysis. D.H. contributed data. C.M.D. conceived and designed the analysis, collected the data, and wrote the manuscript. A.L.L. conceived, designed, and performed the analysis and wrote the manuscript. All authors reviewed the manuscript.

## Conflicts of Interest

The authors have no disclosures related to this manuscript. S.A. receives funding from NIH NIAID and DBV Technologies for contracted work outside of the submitted project; S.A. acknowledges consulting fees with DBV Technologies. C.M.D. receives research grant support from the NIH NIAID (U01 AI126614, U54 AI17804), DBV Technologies, Regeneron Pharmaceuticals, Takeda Pharmaceuticals, and Novartis. The other authors declare that they have no known competing financial interests or personal relationships that could have appeared to influence the work reported in this paper.

## Supporting information


Data S1.

## Data Availability

The data that supports the findings of this study are available in the Supporting Information of this article.
