# Supplementary material for: Shrimp Allergy—Distinct Allergen Sensitization Profiles Between Intercontinental Cohorts
Source: Allergy. 2025 Jul 18;80(10):2909–13. doi: 10.1111/all.16662 (PMC12486353; doi:10.1111/all.16662)
Supplement: Supplementary file 1 — Data S1. [file ALL-80-2909-s001.docx]

**Supplementary**

**Shrimp allergy – Distinct allergen sensitization profiles between intercontinental cohorts**

Shaymaviswanathan Karnaneedi^1,2,3*^, Sara Anvari^4,5^, Shea Brunner^4,5^, Karen S. Tuano^4,5^, Brenda Bin Su^4,5^, Saachi Hira^6^, Sahel Heidari^1,2,3^, Diamond Hira^6^, Carla M. Davis^4,5^, Andreas L. Lopata^1,2,3,7^ *

**Materials and Methods:**

**1. Shrimp Sample Collection, Processing, and Protein Analysis**

Black Tiger shrimp (*Penaeus monodon*) were supplied by the Aquaculture department at James Cook University, Australia, while Vannamei (*Penaeus vannamei*) were purchased from a local supermarket; both arrived frozen. Brown shrimp (*Penaeus aztecus*) were obtained as a powder (F34) from GREER Laboratories, US. For protein extraction, the outer shells of Black Tiger and Vannamei were removed and the edible tissues (abdomen and tail muscle) were cut into small pieces, whereas the Brown shrimp powder was used directly. Protein extraction was conducted as previously described,^1,2^ with some modifications. All samples were homogenized in phosphate-buffered saline (PBS, 4 mL per gram of tissue) using a T10 Basic Ultra-Turrax homogenizer. Heated protein extraction involved heating the homogenate at 95–100°C for 20 minutes, followed by a 16-hour incubation at 4°C with shaking, centrifugation (20,000×g for 25 minutes at 4°C), and vacuum-filtration through a 0.45 μm membrane. Protein concentrations were measured in triplicate using the Pierce™ BCA Protein Assay kit and adjusted to 1 mg/mL. Proteins were separated by SDS-PAGE (Criterion™ system) and visualized by Coomassie Brilliant Blue staining or immunoblotting with patient serum IgE.

**2. Subject Serum, Immunoblotting, and Mass Spectrometry:**

Serum was collected from 15 shellfish-allergic subjects each in Queensland, Australia, and Houston, TX, US (all with positive skin-prick and/or specific IgE tests), plus two non-allergic controls per location, under ethics approval (H4313/H6829). For immunoblotting, proteins were transferred to nitrocellulose membranes, incubated overnight with sera at 4°C, then treated with a mouse anti-human IgE antibody and an infrared-labelled secondary antibody. IgE-binding protein bands were excised, digested with trypsin, and analyzed by mass spectrometry (LC-MS) using Mascot and MaxQuant (iBAQ), as described previously,^3-5^ against a Crustacean database retrieved from UniProt and NCBI Genbank.

**3. Inhibition Immunoblot:**

Inhibition immunoblotting was performed on raw Black Tiger shrimp extract with selected patient sera, using controls and pre-incubation with whole extract or purified allergens (recombinant tropomyosin, natural sarcoplasmic calcium-binding protein, or myosin light chain at 10 and 100 μg/mL).

1. Johnston EB, Kamath SD, Iyer SP, et al. Defining specific allergens for improved component-resolved diagnosis of shrimp allergy in adults. *Molecular Immunology.* 2019;112:330-337.

2. Ruethers T, Taki AC, Karnaneedi S, et al. Expanding the allergen repertoire of salmon and catfish. *Allergy.* 2021;76(5):1443-1453.

3. Nugraha R, Kamath SD, Johnston E, et al. Rapid and comprehensive discovery of unreported shellfish allergens using large-scale transcriptomic and proteomic resources. *Journal of Allergy and Clinical Immunology.* 2018;141(4):1501-1504.e1508.

4. Kamath SD, Liu T, Giacomin P, Loukas A, Navarro S, Lopata AL. Mollusk allergy: Not simply cross-reactivity with crustacean allergens. *Allergy.* 2022;77(10):3127-3130.

5. Kamath SD, Scheiblhofer S, Johnson CM, et al. Effect of structural stability on endolysosomal degradation and T-cell reactivity of major shrimp allergen tropomyosin. *Allergy.* 2020;75(11):2909-2919.


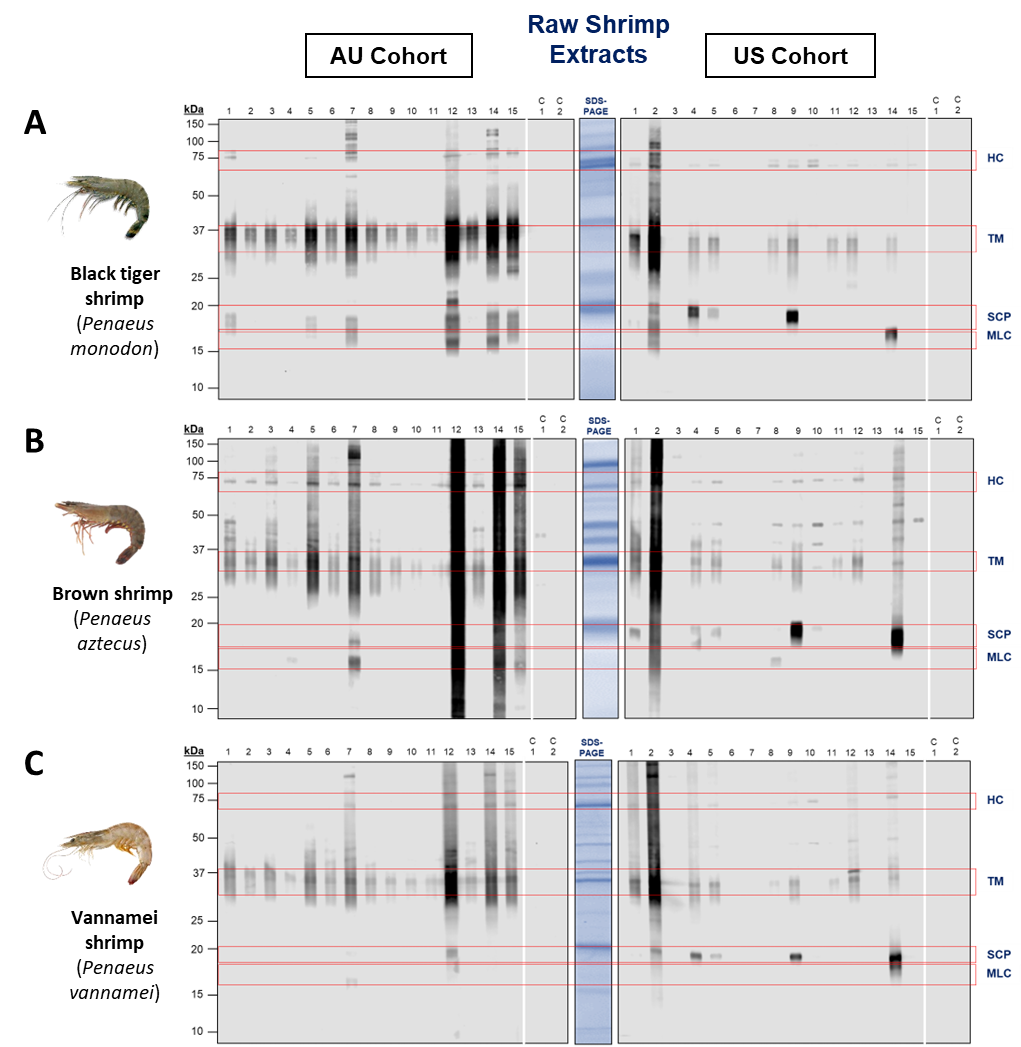


**Figure S1:** IgE immunoblotting: IgE antibody reactivity to **raw shrimp extracts** with sera from shellfish-allergic patient from Australia (AU cohort; *n*=15), the USA (US Cohort; *n*=15), and healthy controls (“C”, *n*=2) against (A) Black Tiger shrimp), (B) Brown shrimp, and (C) Vannamei shrimp. SDS-PAGE of each raw shrimp extract is shown between the immunoblots from different cohorts.


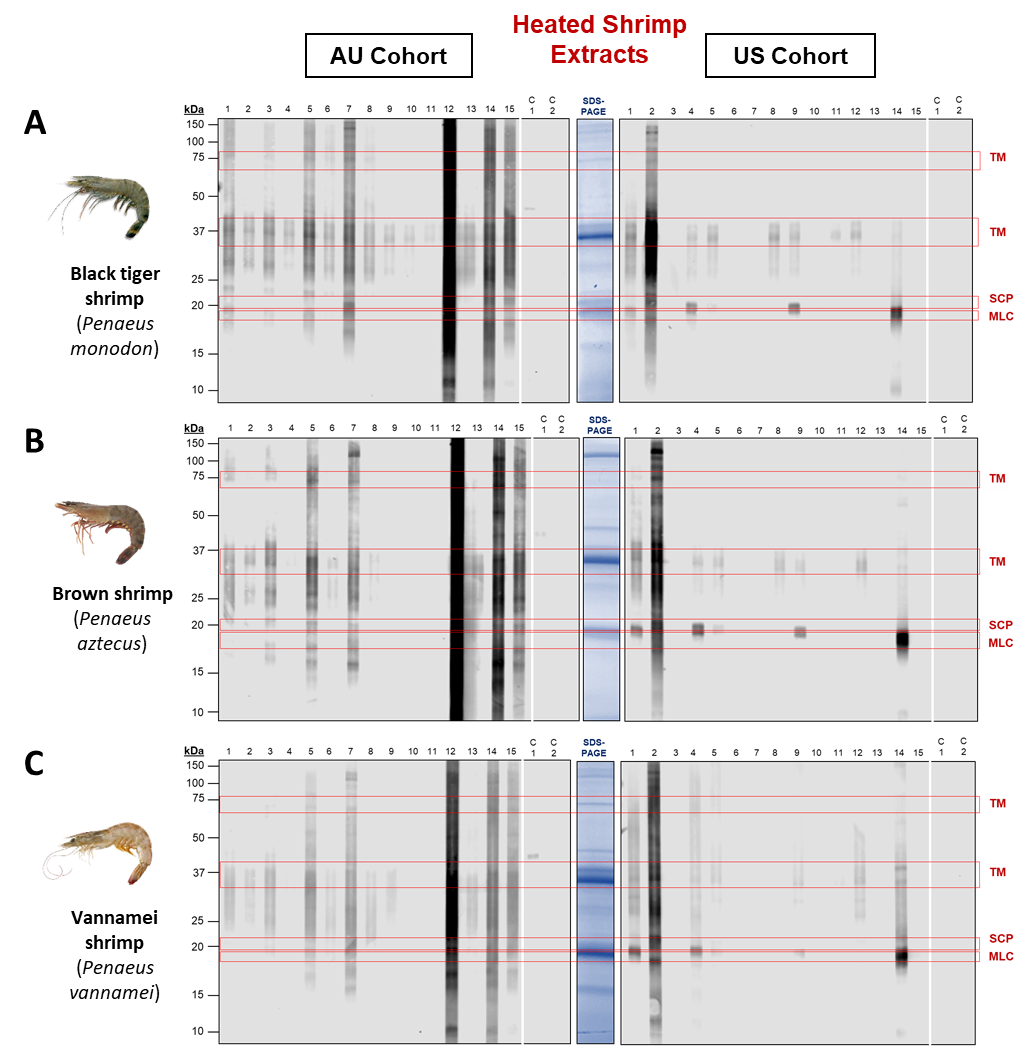


**Figure S2:** IgE immunoblotting: IgE antibody reactivity to **heated/cooked shrimp extracts** with shellfish-allergic patient sera from Australia (AU cohort; *n*=15), the USA (US Cohort; *n*=15), and healthy controls (“C”, *n*=2) against (A) Black Tiger shrimp), (B) Brown shrimp, and (C) Vannamei shrimp. SDS-PAGE separation of each raw shrimp extract is shown between the immunoblots from different cohorts.

**Table S1:** Proteins identified within IgE-binding bands using mass spectrometry (LC-MS/MS).

| **Shrimp Immunoblot** | **MW on SDS-PAGE** | **Identified proteins** | **UniProt/NCBI Genbank Accession Number** | **Known MW of protein (kDa)** | **% Coverage** | **Total peptides** | **iBAQ %** |
| --- | --- | --- | --- | --- | --- | --- | --- |
| **Raw Shrimp Extracts** | | | | | | | |
| **Black tiger shrimp** | 75 | Hemocyanin | XP_037795409.1 | 75 | 32.2 | 33 | 28.9% |
|  | 70 | Hemocyanin | XP_037795419.1 | 75 | 36.3 | 45 | 31.9% |
|  | 40 | Arginine kinase | ACT34086.1 | 40 | 56.2 | 29 | 8.5% |
|  | 35-38 | Tropomyosin | A1KYZ2.1 | 36 | 72.2 | 51 | 47.1% |
|  | 20 | Arginine kinase | ACT34086.2 | 40 | 57.0 | 35 | 16.9% |
|  | 20 | Sarcoplasmic calcium-binding protein | XP_037789865.1 | 20 | 91.7 | 41 | 14.9% |
|  | 18 | Myosin light chain 2 | XP_037801777.1 | 18-20 | 62.2 | 15 | 60.4% |
| **Brown shrimp** | 75 | Hemocyanin | AHY86471.1 | 75 | 26.3 | 28 | 20.2% |
|  | 35-38 | Tropomyosin | A1KYZ2.1 | 36 | 90.8 | 110 | 91.9% |
|  | 20 | Sarcoplasmic calcium-binding protein | ROT72773.1 | 20 | 83.9 | 21 | 69.8% |
|  | 18 | Myosin light chain 2 | ROT76584.1 | 18-20 | 44.2 | 12 | 44.7% |
| **Vannamei shrimp** | 75 | Hemocyanin | AHN85635.1 | 75 | 25.8 | 25 | 2.1% |
|  | 40 | Arginine kinase | ROT68265.1 | 40 | 45.8 | 24 | 53.1% |
|  | 35-38 | Tropomyosin | A1KYZ2.1 | 35-36 | 80.3 | 72 | 87.8% |
|  | 20 | Sarcoplasmic calcium-binding protein | ROT72773.1 | 20 | 67.7 | 11 | 78.8% |
|  | 18 | Myosin light chain 2 | ROT76584.1 | 18-20 | 32.6 | 2 | 2.6% |
| **Heated/Cooked Shrimp Extracts** | | | | | | | |
| **Black tiger shrimp** | 75 | Tropomyosin | A1KYZ2.1 | 36 | 61.6 | 21 | 5.5% |
|  | 35-38 | Tropomyosin | A1KYZ2.1 | 36 | 82.0 | 60 | 93.6% |
|  | 20 | Sarcoplasmic calcium-binding protein | BAL72725.1 | 20 | 71.5 | 21 | 39.4% |
|  | 18 | Myosin light chain 2 | XP_037801777.1 | 18-20 | 62.2 | 14 | 77.6% |
| **Brown shrimp** | 75 | Tropomyosin | A1KYZ2.1 | 36 | 64.1 | 29 | 17.1% |
|  | 35-38 | Tropomyosin | A1KYZ2.1 | 36 | 91.5 | 98 | 91.0% |
|  | 20 | Sarcoplasmic calcium-binding protein | ACM89179.1 | 20 | 67.9 | 17 | 4.5% |
|  | 18 | Myosin light chain 2 | ROT76584.1 | 18-20 | 75.0 | 31 | 80.3% |
| **Vannamei shrimp** | 75 | Tropomyosin | A1KYZ2.1 | 35-36 | 58.8 | 22 | 4.4% |
|  | 35-38 | Tropomyosin | A1KYZ2.1 | 35-36 | 94.4 | 101 | 90.6% |
|  | 20 | Sarcoplasmic calcium-binding protein | ACM89179.1 | 20 | 63.2 | 15 | 2.3% |
|  | 18 | Myosin light chain 2 | ROT76584.1 | 18-20 | 75.0 | 31 | 86.2% |
|  |  |  |  |  |  |  |  |

**Table S2**: Demographics, atopy history, and allergic symptoms of Australian and American shellfish-allergic patient cohort. Note: AR= allergic rhinitis; AD= atopic dermatitis; FA= food allergy; EGID=eosinophilic gastrointestinal disease; GI=Gastrointestinal. p-values indicate the measure of statistical significance (using t-tests) of the difference between the two cohorts. Statistically significant (p < 0.05) differences are indicated in red and non-significant differences in blue.

|  | **Australia** | **USA** | ***p*-values (between cohorts)** |
| --- | --- | --- | --- |
| **Gender**  **Male**  **Female** | 7 (47%)  8 (53%) | 9 (60%)  6 (40%) | *p* = 0.47  *p* = 0.47 |
| **Age mean (years)** | 17 (10-72) | 9 (2-17) | *p* = 0.07 |
| **Age mean of Diagnosis (years)** | 11 (3-21) | 4 (1-8) | *p* = 0.001 |
| **Atopy history**  **Asthma**  **AR**  **AD**  **FA other than shellfish**  **EGID** | 2 (13.3%)  7 (46.7%)  2 (13.3%)  4 (26.7%)  0 (0%) | 9 (60%)  13 (86.7%)  8 (53.3%)  7 (46.7%)  0 (0%) | *p* = 0.008  *p* = 0.02  *p* = 0.02  *p* = 0.25  n.a. |
| **Symptoms**  **Urticaria**  **Angioedema**  **Ocular**  **Respiratory**  **GI** | 8 (53.3%)  12 (80%)  5 (33.3%)  10 (66.7%)  2 (13.3%) | 4 (26.7%)  5 (33.3%)  2 (12.5%)  0 (0%)  0 (0%) | *p* = 0.14  *p* = 0.01  *p* = 0.2  *p* = 0.0001  *p* = 0.14 |


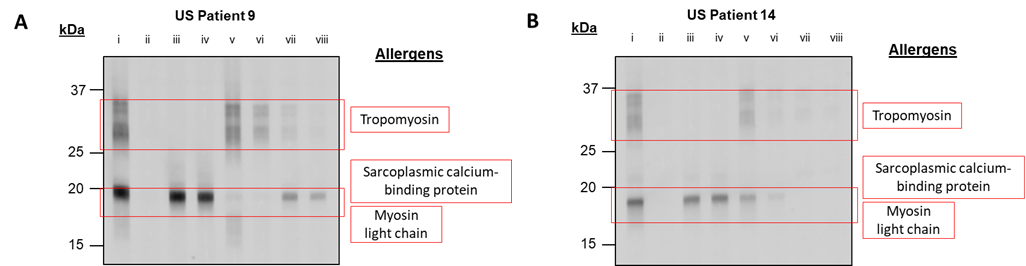


**Figure S3:** Inhibition immunoblot to raw extract of Black Tiger shrimp using serum from (A) US Patient 9 and (B) US Patient 14. Patient serum was pre-incubated with Vannamei shrimp allergens: Lit v 1 tropomyosin at 10 µg/mL (iii), 100 µg/mL (iv), Lit v 4 sarcoplasmic calcium-binding protein at 10 µg/mL (v), 100 µg/mL (vi), Lit v 3 myosin light chain at 10 µg/mL (vii), and 100 µg/mL (viii). No inhibitor (i) was used as the negative control. Inhibition with raw black tiger shrimp extract at 100 µg/mL (ii) was used as the positive control.
